# TREM-1 levels are elevated in patients with liver cirrhosis

**DOI:** 10.1186/cc11958

**Published:** 2013-03-19

**Authors:** SD Gurney, CR Graham, P Kelleher, N Soni, M Foxton, S Singh

**Affiliations:** 1 Chelsea and Westminster Hospital, London, UK; 2 Imperial College, London, UK

## Introduction

Sepsis and spontaneous bacterial peritonitis (SBP) are common sequelae in patients with cirrhosis. Cirrhotics admitted to the ICU have an in-hospital mortality of up to 50% [1]. Microbial translocation (MT) is the pathogenic mechanism implicated in SBP. The triggering receptor expressed by myelocytes-1 (TREM-1) modulates the immune response with resultant production of proinflammatory cytokines and has been used as a biomarker in the diagnosis of bacterial infection. We wish to evaluate the role of TREM-1 as a biomarker in cirrhosis.

## Methods

Blood samples were obtained from 18 healthy controls (HC) and 29 cirrhotic patients (CA) as defined by clinicoradiological criteria. Disease severity was graded according to Child-Pugh class (median 10, range 5 to 13) and modified end-stage liver disease (MELD) score (median 14, range 6 to 21). Simultaneous ascitic fluid samples were taken from 10 patients in the CA group. Soluble TREM-1 and CD14 levels (a surrogate marker of MT) were measured by ELISA. Flow cytometry was used to quantify the expression of TREM-1 on monocytes and neutrophils in blood and ascitic fluid.

## Results

**Figure 1:**
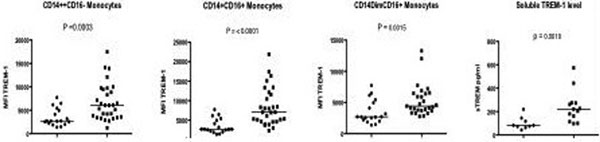
**TREM-1 expression in healthy controls compared with cirrhotic patients**.

TREM-1 expression is significantly higher in the CA group compared with HC across all monocyte subsets but not neutrophils, even in the absence of sepsis (see Figure 1). There is no correlation between cell surface TREM-1 expression and severity of cirrhosis by Child-Pugh or MELD score. sTREM and sCD14 levels were elevated in the CA group compared with HC (*P *= 0.0010 and 0.0016 respectively). In addition, plasma sTREM-1 levels correlated with disease severity according to MELD score (*R *= 0.71, CI = 0.22 to 0.92 *P *= 0.012) and serum bilirubin (*R *= 0.78,CI = 0.36 to 0.94, *P *= 0.004). There was no correlation with either form of TREM-1 with sCD14 levels. There was no difference in cell surface or soluble TREM-1 expression between blood and ascitic fluid monocytes in culture-negative, non-neutrophilic ascites.

## Conclusion

Blood monocyte and soluble TREM-1 are elevated in cirrhotic patients even in the absence of sepsis. Soluble TREM-1 levels correlate with disease severity in cirrhosis. Further studies are ongoing to ascertain the utility of TREM-1 as a biomarker in cirrhosis.

## References

[B1] Olson JC Intensive care of the patient with cirrhosis Hepatology 2011 54 1864 1872 10.1002/hep.24622 21898477

